# Use of POSSUM (Physiologic and Operative Severity Score for the Study of Mortality and Morbidity) and Portsmouth-POSSUM for Surgical Assessment in Patients Undergoing Emergency Abdominal Surgeries

**DOI:** 10.7759/cureus.40850

**Published:** 2023-06-23

**Authors:** Nithya Shekar, PK Debata, Ipsita Debata, Pallavi Nair, Lakshmi S Rao, Prithvi Shekar

**Affiliations:** 1 General Surgery, Vydehi Institute of Medical Sciences and Research Centre, Bengaluru, IND; 2 General Surgery, Kalinga Institute of Medical Sciences, Bhubaneswar, IND; 3 Community and Family Medicine, Kalinga Institute of Medical Sciences, Bhubaneswar, IND; 4 General Surgery, Vydehi Institute of Medical Sciences and Research Center, Bengaluru, IND

**Keywords:** possum, p-possum, morbidity, mortality, abdominal surgery

## Abstract

Introduction: The POSSUM (Physiologic and Operative Severity Score for the Study of Mortality and Morbidity) and Portsmouth-POSSUM (P-POSSUM) models have been popularly recommended as appropriate for predicting postoperative mortality and morbidity in surgical practice. This study aims to evaluate the efficacy and accuracy of both scoring systems for surgical risk assessment in predicting postoperative mortality and morbidity in patients undergoing emergency abdominal surgeries.

Methodology: The study was conducted as a part of a post-doctoral fellowship program. A total of 150 patients, undergoing emergency abdominal surgery in a tertiary care hospital in Bhubaneswar, were evaluated using POSSUM and P-POSSUM. Physiological scoring was done prior to surgery and operative scoring was performed intra-operatively. Patients were followed up for 30 days after the operative period. The observed mortality rate was then compared with POSSUM and P-POSSUM predicted mortality rates.

Results: POSSUM predicted a morbidity rate of 116, whereas the actual morbidity rate was 92 (p < 0.05). P-POSSUM predicted a morbidity rate of 109, whereas the actual morbidity rate was 92 (p < 0.05). POSSUM predicted a mortality rate of 23, whereas the actual mortality rate was 21 (p < 0.05). P-POSSUM predicted a mortality rate of 25, whereas the actual mortality rate was 21 (p < 0.05).

Conclusions: With a reasonably good prediction of morbidity and mortality rate, POSSUM and P-POSSUM scores are both effective scoring systems in clinical practice for use in abdominal surgery.

## Introduction

Millions of people, worldwide, require emergency abdominal surgery for potential catastrophic small bowel obstructions, gastrointestinal tract perforations, hemorrhages, invasive cancerous tumors, blunt force/penetrative trauma injuries, and peritonitis every year. Emergency surgery constitutes approximately 11% of total surgical cases in developed countries and yet surprisingly can contribute almost half toward surgical mortality and a third toward surgical complications. Postoperative outcomes following emergency abdominal surgery are generally inferior when compared to elective surgery. The most common complication after emergency abdominal surgery is a postoperative pulmonary complication with an incidence rate of 20-50% [1].

The prevalence of gastrointestinal emergencies in tropical countries significantly differs from that in temperate countries. Penetrating abdominal injury has also become a common reason for emergency admission. Emergency gastrointestinal surgeries have considerably higher mortality and morbidity rates as compared to elective surgery [2].

Although being one of the most common urgent surgical procedures in India, there is a scarcity of data concerning indications and postoperative mortality rates after emergency laparotomy [3].

Crude morbidity and mortality rates are misleading because these do not account for the demographic and physiological parameters of the patient at the time of surgery. For a meaningful comparison, some risk-adjusted analysis must be performed [4].

The outcome of all surgeries performed not only depends on the surgeon's performance but also on the patient's clinical status at the time of surgery. There has been a need to develop a system that can predict the outcome of emergency abdominal surgery, with a priority on developing scoring systems that can standardize patient data to enable meaningful comparisons [5].

Several predictors of morbidity and mortality are available. However, POSSUM (Physiological and Operative Severity Score for the Enumeration of Mortality and Morbidity) has been recommended as appropriate for surgical practice. The system aims to predict mortality and morbidity in the initial 30 postoperative days and allows comparison of results within the institution over time or performs a cross-sectional comparative analysis with other institutions [6]. The POSSUM score assesses expected morbidity and mortality, adjusted for risk in a given context [7].

The POSSUM scoring system has been found to overestimate mortality, especially in patients with low risk. To address this problem across a number of surgical procedures, modifications of the POSSUM scoring system have been proposed, such as Portsmouth-POSSUM (P-POSSUM) and oesophagogastric-POSSUM (o-POSSUM). Numerous researchers have reported that the predictive ability of P-POSSUM is superior and more accurate when compared to POSSUM [8]. It has the same grading system and variables, but a different equation, providing a better fit to the observed mortality rate, which is an objective measure of the outcome. It has already been used in general, colorectal, vascular, esophageal, and laparoscopic procedures, but the studies have mostly involved patients in developed countries, where the characteristics and presentation of patients and available resources differ from developing countries [9].

There is a need to evaluate if the P-POSSUM scoring system can effectively and accurately address these concerns while estimating the expected mortality rate in Indian clinical settings [10]. It would be logical to include the major elective and emergency surgeries, previously defined by the POSSUM scoring system because this includes patients belonging to high-risk groups where the comparison between observed and expected mortality rates can produce significant results and help determine the possible causes for unfavorable outcomes [11]. Many scores have been devised that are ideally suited to special types of surgical procedures or to assess particular types of complications. In the present study, POSSUM and P-POSSUM scoring systems were applied to determine their accuracy in predicting morbidity and mortality in patients undergoing abdominal surgery.

## Materials and methods

Study setting and duration

A hospital-based observational study was carried out for two years in the Department of Surgery at Kalinga Institute of Medical Sciences (KIMS), Bhubaneswar, as a part of the post-doctoral residency program. Ethical clearance was obtained from the Institutional Ethical Committee (KIIT/KIMS/IEC/145/2019) and the protocol followed the principles of the Declaration of Helsinki.

Sample size

The study was carried out among 150 patients who were admitted to the surgery department of the tertiary care hospital and who underwent emergency abdominal surgery, from September 2019 to August 2021, with a 30-day follow-up period. The universal sampling method was followed to include the participants satisfying the inclusion and exclusion criteria.

Inclusion criteria

The inclusion criteria were (a) patients who underwent emergency abdominal surgeries at KIMS hospital during the study period, (b) patients who provided informed consent for the study, and (c) patients who agreed to a follow-up evaluation 30 days after surgery.

Exclusion criteria

The exclusion criteria were (a) patients younger than 18 years and (b) patients who were immune suppressed (human immunodeficiency virus [HIV]/hepatitis B virus surface antigen [HBsAg]/hepatitis C virus [HCV] positive, on immunosuppressive drugs, or on anti-cancer chemotherapeutic drugs). All participating patients and their family members provided written informed consent.

Data collection

Socio-demographic details, clinical examination findings, lab parameters, and chest X-ray findings were collected with a predesigned and semi-structured questionnaire.

POSSUM and P-POSSUM

POSSUM included two types of scores, six operative severity scores (OS) and 12 physiology scores (PS). Each component was classified based on increasing scores (1, 2, 4, and 8) (Table 1). The assigned score was 1 in case the data was missing [12,13]. The scoring criteria are shown in Table 1. Physiological scoring was calculated prior to surgery and the operative scoring was calculated during or intra-operatively. Patients were followed up for the first 30 days of the postoperative period. By substituting PS and OS into regression equations, the POSSUM scoring system predicted the postoperative complication rate (R1) and mortality rate (R2), and the P-POSSUM predicted the postoperative mortality rate (R). The calculation equation [12, 14] was as follows:

lnR1 / (1 - R1) = -5.91 + 0.16 * PS + 0.19 * OS

lnR2 / (1 - R2) = -7.04 + 0.13 * PS + 0.16 * OS

lnR / (1 - R) = -0.065 + 0.1692 * PS + 0.1550 * OS

**Table 1:** Parameters to calculate POSSUM score POSSUM, physiological and operative severity score for the enumeration of mortality and morbidity; JVP, jugular venous pressure.

Parameters	Physiological score			
	1	2	4	8
Age (in years)	≤ 60	61-70	≥71	
Cardiac signs	No failure	Diuretic, digoxin, anti-anginal, or antihypertensive therapy	Peripheral edema, warfarin therapy, borderline cardiomegaly	Raised JVP, cardiomegaly
Respiratory signs	No dyspnea	Dyspnea on exertion, mild COPD	Limiting dyspnea (one flight), moderate/COPD	Dyspnea at rest (rate ≥30/min), fibrosis, or consolidation
Blood pressure (systolic in mmHg)	110- 130	131-170; 100-109	≥171; 90-99	≤89
Pulse (beats/min)	50-80	81-100; 40-49	101-120	≥120; ≤39
Glasgow coma scale	15	12-14	9-11	≤8
Hemoglobin	13-16	11.5-12.9; 16.1-17.0	10.0-11.4; 17.1-18.0	≤9.9; ≥18.1
White cell count (× 10^12^/L)	4-10	10.1-20.0; 3.1-4.0	≥20.1; ≤3.0	
Urea (mg/dL)	≤40	41-55	55-80	≥80
Sodium (mmol/L)	≥136	131-135	126-130	≤125
Potassium (mmol/L)	3.5-5.0	3.2-3.4; 5.1-5.3	2.9-3.1; 5.4-5.9	≥6.0
ECG	Normal		Atrial fibrillation	Any abnormal rhythm or ≥5 ectopics/min, Q waves or ST/T wave changes
	Operative severity score			
	1	2	4	8
Operative severity	Minor	Moderate	Major	Major+
Multiple procedures	1		2	>2
Total blood loss (mL)	≤100	101-500	501-999	≥1000
Peritoneal soiling	None	Minor (serous fluid)	Local pus	Free bowel content (pus or blood)
Presence of malignancy	None	Primary only	Nodal metastases	Distant metastases
Mode of surgery			Emergency resuscitation or >2 h possible operation within 24 hours after admission	Emergency (immediate surgery <2 h needed)

PS and OS of each patient were substituted into the regression equations above, and the complication risk coefficient and mortality risk coefficient were obtained, which were predicted by POSSUM and P-POSSUM. These risk coefficients were then converted into percentages. These predicted complication rates and mortality rates were then used to derive the overall predicted complication rate and mortality rate of 150 patients.

Statistical analysis

Data obtained were entered into Excel and analyzed using linear regression analysis with SPSS version 21 (Armonk, NY: IBM Corp). The observed:expected (O-E) ratio was estimated to obtain the expected mortality rate. The association between the POSSUM score and poor outcome and the P-POSSUM score and poor outcome was evaluated by the chi-square (χ^2^) test. The rate of increase in mortality for each risk factor was calculated based on the hypothesis that deaths were linearly related to the score for each of the risk factors under evaluation.

## Results

A total of 150 patients were included in the study; 109 (73%) were males and 41 (27%) were females. A total of 43% of patients were between 31 and 40 years of age (Table 2). The mortality of our study was 19% (21).

**Table 2:** Distribution of patients according to age

Intervals	Frequency (n = 150)	Percentage
21-30	42	28
31-40	64	43
41-50	28	19
51-60	13	8
61-70	3	2

Peritoneal soiling

More than half (51%) of the patients had bile as their peritoneal contents (Table 3).

**Table 3:** Distribution of patients according to peritoneal contents

Peritoneal contents	Frequency (n = 150)	Percentage
Bile	76	51
Feces	17	11
Blood	24	16
Serous fluid	8	5
Pus	3	2
Nil	22	15

Physiological score

**Figure 1:**
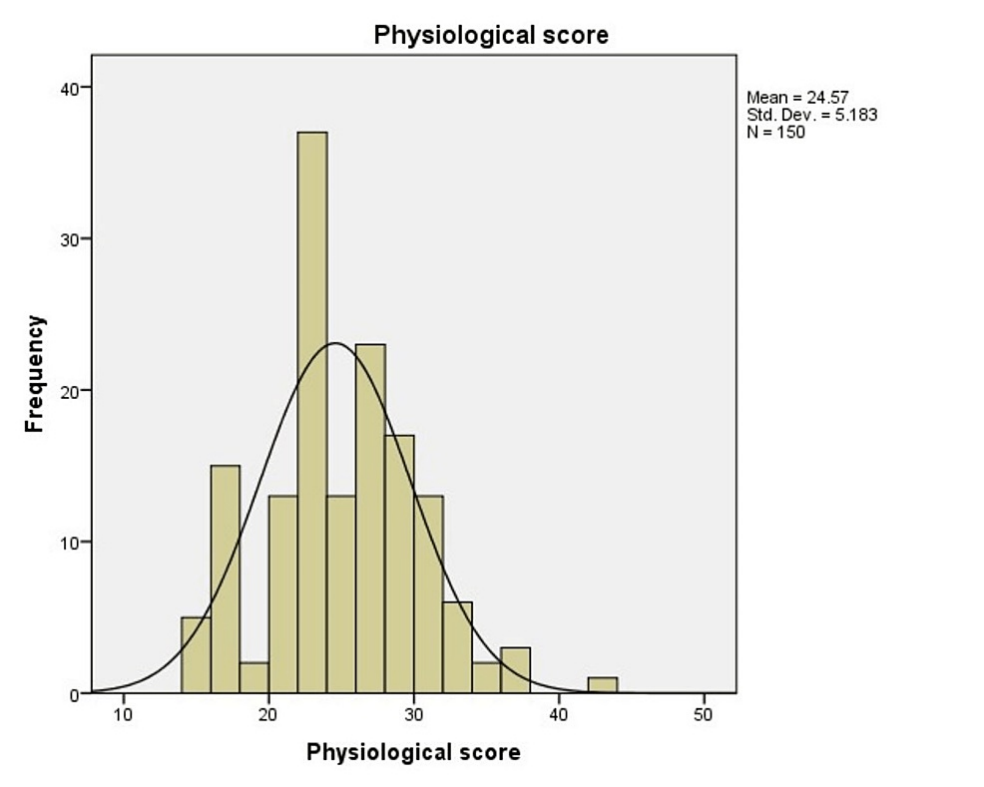
Histogram showing the distribution of patients according to the physiological score

The physiological scores ranged from 15 to 43, with a mean score of 24.57. A total of 42% (63) patients had a physiological score between 21 and 25, 34% (51) of patients had a physiological score of 26-30, 14.67% (22) patients had a physiological score of 15-20, whereas only 0.67% (1) patients had a physiological score of 41-45 (Table 4; Figure 1).

**Table 4:** Distribution of patients according to physiological score

Physiological score	Frequency (n = 150)	Percentage
15-20	22	14.67
21-25	63	42
26-30	51	34
31-35	10	6.67
36-40	3	2
41-45	1	0.67

59.33% (89) subjects had an operative score of 16-20 and 19.33% (29) subjects had an operative score of 10-15, whereas only 8% (12) subjects had an operative score of 26-30 (Table 5).

**Table 5:** Distribution of patients according to operative score

Operative score	Frequency (n = 150)	Percentage
10-15	29	19.33
16-20	89	59.33
21-25	20	13.33
26-30	12	8

**Figure 2:**
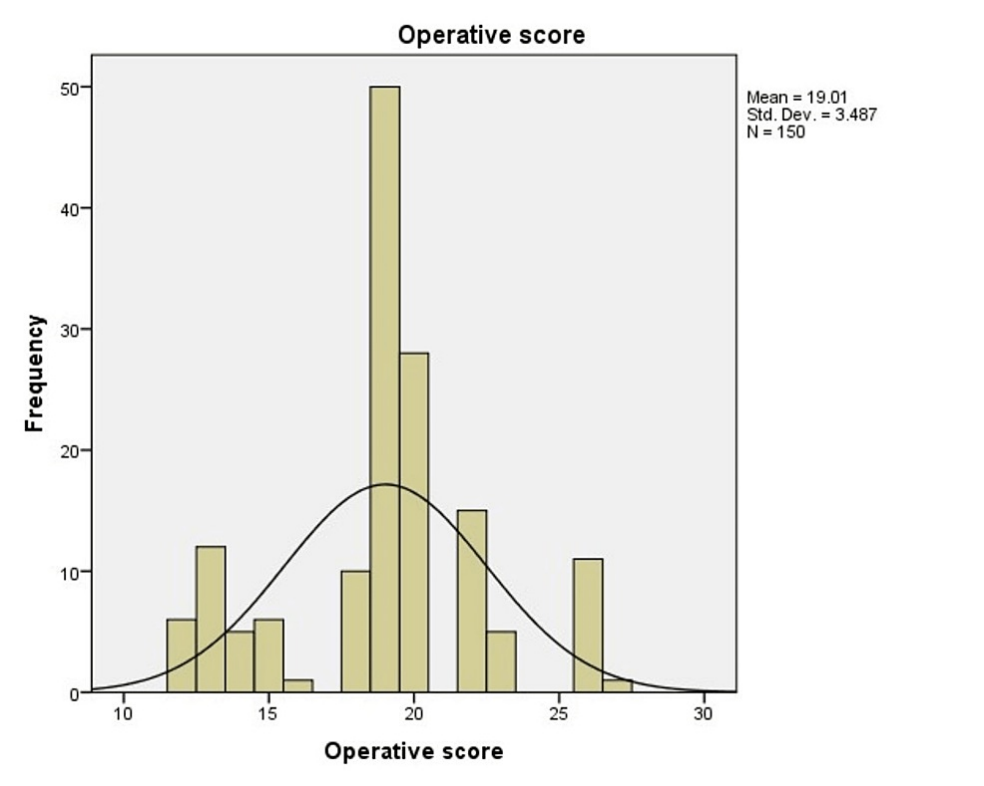
Histogram showing the distribution of patients according to the operative score

The physiological scores from our study group were more skewed toward the higher side because of low hemoglobin levels. The operative scores varied from 12 to 30 with a mean of 19.01. Most of our patients had peritoneal contamination, which presented in the form of biliary, fecal contamination or pus collections, elevating the operative scores and resulting in an increase in the number of predicted deaths (Figure 2).

The number of morbidities predicted by POSSUM was 116, whereas the actual observed number of morbidities was 92. The difference was statistically significant (p < 0.05) (Table 6). 

**Table 6:** Comparison of morbidity predicted by POSSUM scoring to actual morbidity POSSUM, Physiologic and Operative Severity Score for the Study of Mortality and Morbidity.

POSSUM predicted morbidity	Number of patients	Predicted number of morbidity	Observed number of morbidity	Observed:Expected
0.00-0.10	0	0	0	0
0.11-0.20	0	0	0	0
0.21-0.30	0	0	0	0
0.31-0.40	0	0	0	0
0.41-0.50	0	0	0	0
0.51-0.60	8	5	4	0.8
0.61-0.70	29	19	10	0.53
0.71-0.80	53	38	33	0.87
0.81-0.90	59	53	44	0.83
0.91-1.00	1	1	1	100
0.00-1.00	150	116	92	0.79

**Figure 3:**
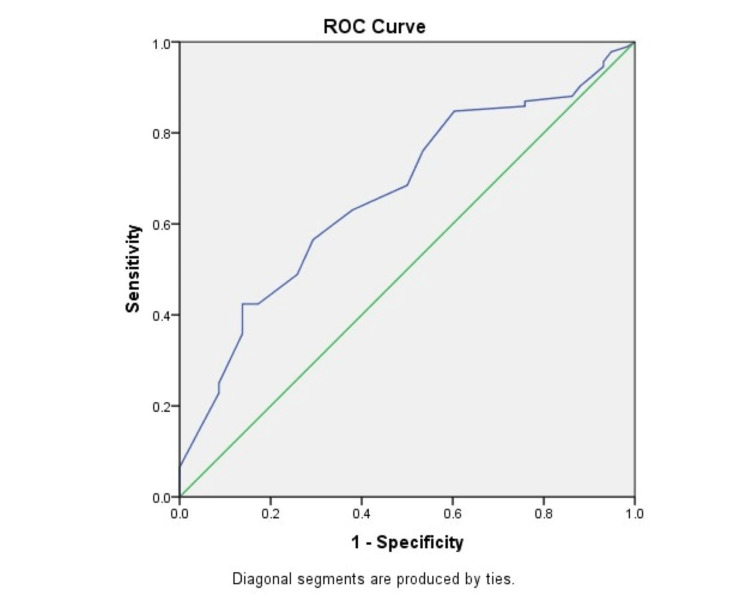
ROC curve showing morbidity predicted by POSSUM scoring as compared to actual morbidity ROC, receiver operating characteristic; POSSUM, Physiologic and Operative Severity Score for the Study of Mortality and Morbidity.

Figure 3 shows a receiver operating characteristic (ROC) curve showing morbidity predicted by POSSUM scoring as compared to actual morbidity. The area under the curve was 0.666, which showed moderate prediction ability of the POSSUM score for morbidity.

The number of morbidities predicted by P-POSSUM was 109, whereas the actual observed number of morbidities was 92. The difference was statistically significant (p < 0.05) (Table 7).

**Table 7:** Comparing morbidity predicted by P-POSSUM scoring to actual morbidity P-POSSUM, Portsmouth-POSSUM; POSSUM, Physiological and Operative Severity Score for the Enumeration of Mortality and Morbidity.

P-POSSUM predicted morbidity	Number of patients	Predicted number of morbidity	Observed number of morbidity	Observed:Expected
0.00-0.10	0	0	0	0
0.11-0.20	0	0	0	0
0.21-0.30	0	0	0	0
0.31-0.40	0	0	0	0
0.41-0.50	1	1	0	0
0.51-0.60	8	5	5	1
0.61-0.70	50	33	24	0.73
0.71-0.80	52	37	32	0.86
0.81-0.90	39	33	31	0.94
0.91-1.00	0	0	0	0
0.00-1.00	150	109	92	0.84

**Figure 4:**
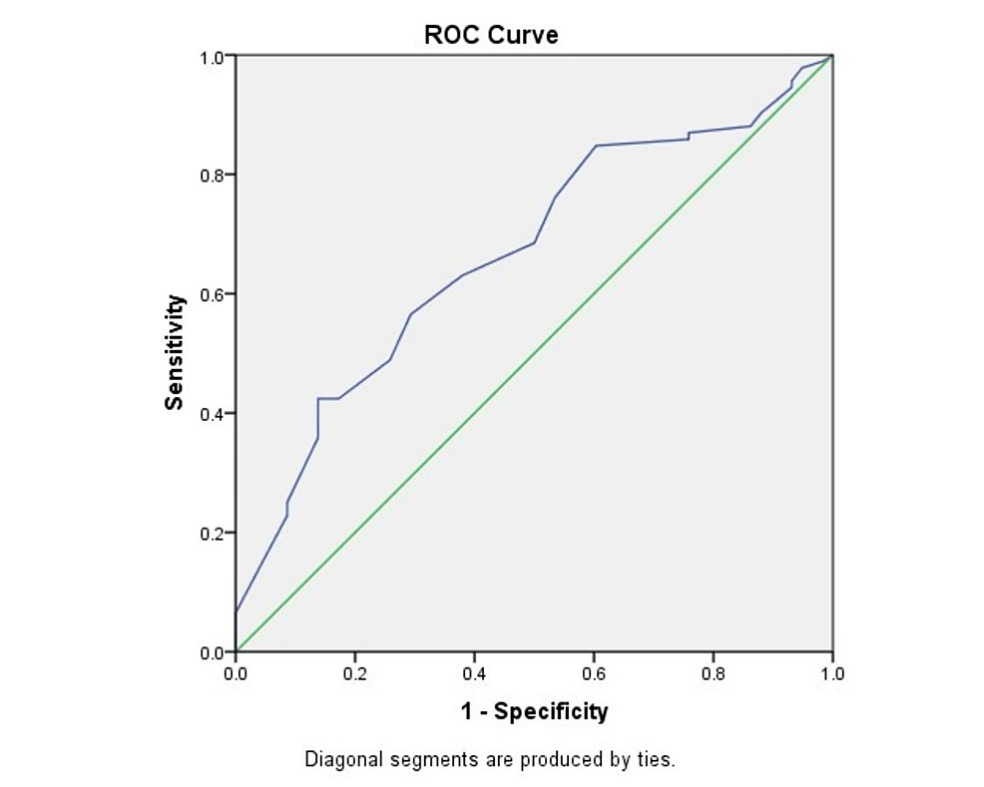
ROC curve showing morbidity predicted by P-POSSUM scoring as compared to actual morbidity ROC, receiver operating characteristic; P-POSSUM, Portsmouth-POSSUM; POSSUM, Physiological and Operative Severity Score for the Enumeration of Mortality and Morbidity.

Figure 4 shows an ROC curve showing morbidity predicted by P-POSSUM scoring as compared to actual morbidity. The area under the curve was 0.664, which showed a moderate prediction ability of the P-POSSUM score for morbidity.

The number of deaths predicted by POSSUM was 23, whereas the actual observed number of deaths was 21. The difference was statistically significant (p < 0.05) (Table 8).

**Table 8:** Comparing mortality predicted by POSSUM scoring to actual mortality POSSUM, Physiological and Operative Severity Score for the Enumeration of Mortality and Morbidity.

POSSUM predicted mortality	Number of patients	Predicted number of deaths	Observed number of deaths	Observed:Expected
0.00-0.10	9	1	0	0
0.11-0.20	36	5	1	0.2
0.21-0.30	23	3	0	0
0.31-0.40	33	5	2	0.4
0.41-0.50	32	5	9	1.8
0.51-0.60	8	1	5	5
0.61-0.70	7	1	2	2
0.71-0.80	1	1	1	1
0.81-0.90	1	1	1	1
0.91-1.00	0	0	0	0
0.00-1.00	150	23	21	0.91

**Figure 5:**
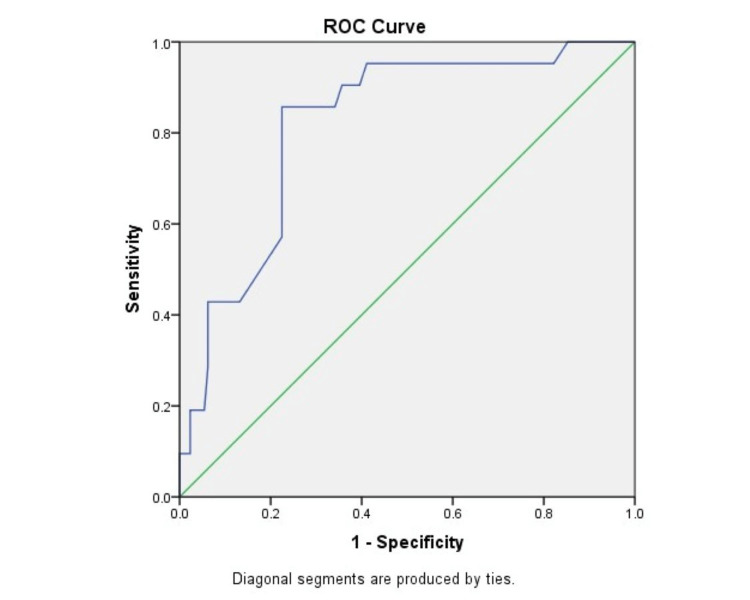
ROC curve showing mortality predicted by POSSUM scoring as compared to actual mortality ROC, receiver operating characteristic; POSSUM, Physiologic and Operative Severity Score for the Study of Mortality and Morbidity.

Figure 5 shows an ROC curve showing mortality predicted by POSSUM scoring as compared to actual mortality. The area under the curve was 0.818, which showed a moderate prediction ability of POSSUM scoring for mortality.

The number of deaths predicted by P-POSSUM was 25, whereas the actual observed number of deaths was 21. The difference was statistically significant (p < 0.05) (Table 9).

**Table 9:** Comparing mortality predicted by P-POSSUM scoring to actual mortality P-POSSUM, Portsmouth-POSSUM; POSSUM, Physiological and Operative Severity Score for the Enumeration of Mortality and Morbidity.

P-POSSUM predicted mortality	Number of patients	Predicted number of deaths	Observed number of deaths	Observed:Expected
0.00-0.10	63	9	1	0.11
0.11-0.20	39	6	4	0.67
0.21-0.30	19	3	4	1.33
0.31-0.40	21	3	9	3
0.41-0.50	3	1	1	1
0.51-0.60	3	1	0	0
0.61-0.70	1	1	1	1
0.71-0.80	1	1	1	1
0.81-0.90	0	0	0	0
0.91-1.00	0	0	0	0
0.00-1.00	150	25	21	0.84

**Figure 6:**
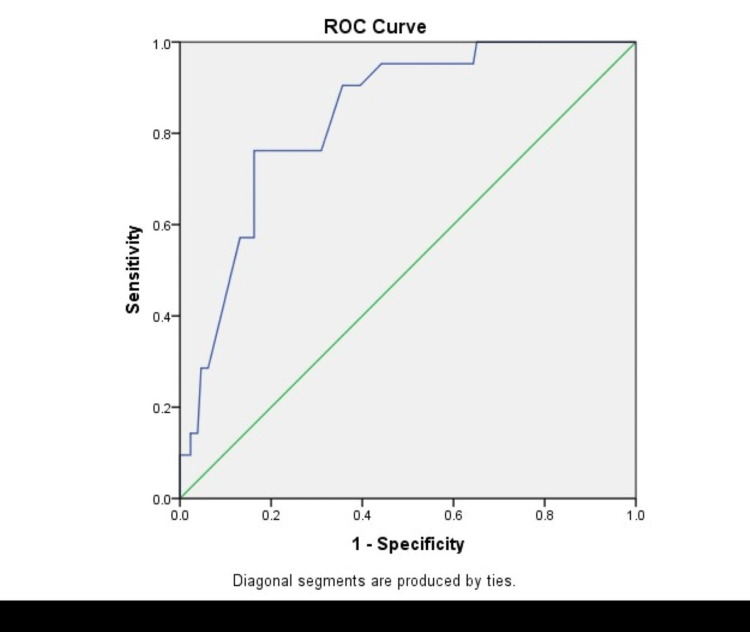
ROC curve showing mortality predicted by P-POSSUM scoring as compared to actual mortality ROC, receiver operating characteristic; P-POSSUM, Portsmouth-POSSUM; POSSUM, Physiological and Operative Severity Score for the Enumeration of Mortality and Morbidity.

Figure 6 shows an ROC curve showing mortality predicted by P-POSSUM scoring compared to actual mortality. The area under the curve was 0.836, which showed a moderate prediction ability of P-POSSUM scoring for mortality.

## Discussion

Emergency laparotomy “describes an exploratory procedure for which the clinical presentation, underlying pathology, anatomical site of surgery, and perioperative management vary considerably” [15]. Evaluating the risk of surgery accurately, based on physiological and operative parameters could help decide the best specific treatment for patients according to the estimated risk [16,17].

The POSSUM scoring system can predict morbidity and mortality 30 days after surgery mathematically by regression analysis. Because it overestimated the predicted mortality, P-POSSUM was developed, which provided a more apt fit to the observed mortality rate [10]. It has been widely used for vascular, colorectal, general, laparoscopic, and esophageal procedures [18-25]. However, these studies have mostly been conducted in Western countries where patients differ in presentation, characteristics, and hospital resource settings.

In the present study, 43% of patients were in between 31 and 40 years of age and 28% were between 21 and 30 years, whereas only 2% were between 61 and 70 years of age. Patients' ages ranged from 21 to 66 years, with a mean age of 37 years. In a study by Sergio González-Martínez et al. [7], the mean age of patients included was 59.2 years. In a study by Avinash Vishwani et al. [5], 25.8% belonged to the age group 21-30, followed by 19.1% in the age group 51-60 years. A study by Dilip Kumar Das et al. [9] reported the average age of the study participants as 40.6 ± 16.67. The mean age was 75.4 ± 7.3 years in a study by Yang Cao et al. [25]. Out of 150 patients, 41 were females and 109 were males. The male-to-female sex ratio was around 1.9:1 in a study by Singh et al. [26].

In the present study, the mortality rate was 19%. In a study by Amarnath Kumar et al. [27], out of 50 patients studied, there was a crude mortality rate of 18%. Of the 41 alive patients, 25 had at least one complication, amounting to 61% of the crude morbidity rate. A study by Sergio González-Martínez et al. [7] reported an overall mortality rate of 2.1%. In the present study, the physiological scores ranged from 15 to 43, with a mean score of 24.57. A similar mean physiological score of 23.65 was reported by Rebecka Ahl et al. [28]. The physiological score alone cannot be used to predict the risk of the development of complications because of confounding factors [29,30]. In the present study, the operative scores ranged from 12 to 30, with 19.01 as the mean score. Many patients had peritoneal contamination in the form of biliary, fecal, or pus collections, which elevated the operative scores and hence increased the number of predicted deaths. Similar scores were reported by other studies in other parts of the world [7,29,30]. A study by Dilip Kumar Das et al. [9] recorded that the operative severity score (p = 0.017) was significantly associated with mortality in the study population. Operative scores in P-POSSUM had a greater effect on the mortality rate than the operative scores of POSSUM. This could be due to the greater number of parameters measured in the P-POSSUM operative score affecting the postoperative period. The need to optimize the patient physiologically cannot be overstressed.

In comparison between the two scoring systems, it was found in our study that the observed-to-expected morbidity and mortality ratios were 0.79 and 0.91, respectively, by POSSUM and 0.84 and 0.84, respectively, by P-POSSUM. Both the POSSUM and P-POSSUM predicted morbidity and mortality rates were more than the observed ones and were statistically significant (p < 0.05). However, in a study by Amarnath Kumar et al. [27], observed-to-expected mortality and morbidity ratios were 1.005 and 1.001, respectively, and there was no statistically significant difference between the observed and predicted values. Similar findings were noted in other studies [4,7]. We also estimated the area under the ROC curve. The area under the ROC curve was 0.666 and 0.664 for morbidity prediction by POSSUM and P-POSSUM, respectively, thus showing moderate accuracy. The area under the ROC curve was 0.818 and 0.836 for mortality prediction by POSSUM and P-POSSUM, respectively, thus showing the accuracy to be higher. The results show that POSSUM and P-POSSUM can accurately predict postoperative complications, with mortality prediction better at this than morbidity, in patients with emergency abdominal surgeries. The findings are consistent with findings from other studies [31,32].

Limitations of our study

Our sample size was small, due to which certain data evaluations could not be carried out. This was a single-center study. Further large-scale research with multiple centers is required to validate the accuracy of the scores and to substantiate our claim. Even though we followed up with all discharged patients for 30 days postoperatively at outpatient clinics, some patients with complications may not have been discovered.

## Conclusions

We can conclude that POSSUM and P-POSSUM scores have a moderate ability to predict mortality and morbidity rates in emergency abdominal surgery patients. Both the scores had an almost equal ability of prediction, with P-POSSUM having a higher accuracy for mortality prediction. Both can be used to predict surgical outcomes in emergency abdominal surgery cases.

## References

[1] Boden I, Sullivan K, Hackett C, Winzer B, Lane R, McKinnon M, Robertson I (2018). ICEAGE (Incidence of Complications following Emergency Abdominal surgery: Get Exercising): study protocol of a pragmatic, multicentre, randomised controlled trial testing physiotherapy for the prevention of complications and improved physical recovery after emergency abdominal surgery. World J Emerg Surg.

[2] Hota PK, Yellapragada H (2017). Assessment of surgical outcome in emergency gastrointestinal surgeries using P-POSSUM score. Int J Res Med Sci.

[3] Parray AM, Mwendwa P, Mehrotra S (2018). A review of 2255 emergency abdominal operations performed over 17 years (1996-2013) in a gastrointestinal surgery unit in India. Indian J Surg.

[4] Yadav K, Singh M, Griwan M, Mishra Ts, Kumar N, Kumar H (2011). Evaluation of POSSUM and P-POSSUM as a tool for prediction of surgical outcomes in the Indian population. Australas Med J.

[5] Vishwani A, Gaikwad VV, Kulkarni RM, Murchite S (2014). Efficacy of Possum scoring system in predicting mortality and morbidity in patients of peritonitis undergoing laparotomy. Int J Sci Study.

[6] Carvalho-E-Carvalho ME, De-Queiroz FL, Martins-Da-Costa BX, Werneck-Côrtes MG, Pires-Rodrigues V (2018). The applicability of POSSUM and P-POSSUM scores as predictors of morbidity and mortality in colorectal surgery. Rev Col Bras Cir.

[7] González-Martínez S, Martín-Baranera M, Martí-Saurí I, Borrell-Grau N, Pueyo-Zurdo JM (2016). Comparison of the risk prediction systems POSSUM and P-POSSUM with the Surgical Risk Scale: A prospective cohort study of 721 patients. Int J Surg.

[8] Hong S, Wang S, Xu G, Liu J (2017). Evaluation of the POSSUM, p-POSSUM, o-POSSUM, and APACHE II scoring systems in predicting postoperative mortality and morbidity in gastric cancer patients. Asian J Surg.

[9] Das DK, Singh AK, Roy S, Mukherjee R, Samanta S (2019). Evaluation of clinical outcome of patients undergoing emergency laparotomy with the help of Portsmouth predictor equation for mortality (P-Possum score). Int J Contemp Med.

[10] Tonape T, Suma R, Jaiswal R, Kelshikar S, Parekh R (2022). The study of evaluation of Portsmouth Possum scoring in predicting mortality and morbidity and also identifying risk factors for low outcome in abdominal aurgeries. J Pharm Negat Results.

[11] Copeland GP, Jones D, Walters M (1991). POSSUM: a scoring system for surgical audit. Br J Surg.

[12] Lima DF, Cristelo D, Reis P, Abelha F, Mourão J (2019). Outcome prediction with Physiological and Operative Severity Score for the enumeration of Mortality and Morbidity score system in elderly patients submitted to elective surgery. Saudi J Anaesth.

[13] Whiteley MS, Prytherch DR, Higgins B, Weaver PC, Prout WG (1996). An evaluation of the POSSUM surgical scoring system. Br J Surg.

[14] Nag DS, Dembla A, Mahanty PR, Kant S, Chatterjee A, Samaddar DP, Chugh P (2019). Comparative analysis of APACHE-II and P-POSSUM scoring systems in predicting postoperative mortality in patients undergoing emergency laparotomy. World J Clin Cases.

[15] Teixeira IM, Teles AR, Castro JM, Azevedo LF, Mourão JB (2018). Physiological and Operative Severity Score for the enumeration of Mortality and Morbidity (POSSUM) system for outcome prediction in elderly patients undergoing major vascular surgery. J Cardiothorac Vasc Anesth.

[16] Sharrock AE, McLachlan J, Chambers R, Bailey IS, Kirkby-Bott J (2017). Emergency abdominal surgery in the elderly: Can we predict mortality?. World J Surg.

[17] Manivannan DR, Prabhakaran DM (2016). Evaluation of POSSUM scoring in patients undergoing emergency laparotomy for hollow viscus perforation. IOSR J Pharm Biol Sci.

[18] Bertleff MJ, Lange JF (2010). Perforated peptic ulcer disease: a review of history and treatment. Dig Surg.

[19] Brian W, Ellis SP (2012). Hamilton Bailey’s Emergency Surgery, 13th ed. pp. 307-326.

[20] Moran B, Hollingshead J, Farquharson M (2015). Farquharson’s Textbook of Operative General Surgery, 10th ed. https://books.google.co.in/books?id=JtEgCAAAQBAJ&printsec=frontcover&source=gbs_ge_summary_r&cad=0#v=onepage&q&f=false.

[21] Thorsen K, Søreide JA, Søreide K (2013). Scoring systems for outcome prediction in patients with perforated peptic ulcer. Scand J Trauma Resusc Emerg Med.

[22] Gawande AA, Kwaan MR, Regenbogen SE, Lipsitz SA, Zinner MJ (2007). An Apgar score for surgery. J Am Coll Surg.

[23] Miyazaki N, Haga Y, Matsukawa H, Ishimura T, Fujita M, Ejima T, Tanimoto H (2014). Development and validation of the Calculation of post-Operative Risk in Emergency Surgery (CORES) model. Surg Today.

[24] Kennedy RH, al-Mufti RA, Brewster SF, Sherry EN, Magee TR, Irvin TT (1994). The acute surgical admission: Is mortality predictable in the elderly?. Ann R Coll Surg Engl.

[25] Cao Y, Bass GA, Ahl R, Pourlotfi A, Geijer H, Montgomery S, Mohseni S (2020). The statistical importance of P-POSSUM scores for predicting mortality after emergency laparotomy in geriatric patients. BMC Med Inform Decis Mak.

[26] Singh TK, Devi SR, Maibam C, Raut NR, Singh J (2013). Portsmouth physiological and operative severity score for the enumeration of mortality and morbidity scoring system in general surgical practice and identifying risk factors for low outcome. J Med Soc.

[27] Kumar A, Suman S, Kundan K, Kumar P (2016). Evaluation of POSSUM scoring system in patients with perforation peritonitis. Int Surg J.

[28] Ah R, BChir MB, Cao Y (2019). Prognostic value of P-POSSUM and osteopenia for predicting mortality after emergency laparotomy in geriatric patients. Bull Emerg Trauma.

[29] Kitara DL, Kakande I, Mugisa BD (2007). POSSUM scoring system in patients undergoing laparotomy in Mulago Hospital. East Cent Afr J Surg.

[30] Moonesinghe SR, Mythen MG, Das P, Rowan KM, Grocott MP (2013). Risk stratification tools for predicting morbidity and mortality in adult patients undergoing major surgery: Qualitative systematic review. Anesthesiology.

[31] Chatterjee AS, Renganathan DN (2015). POSSUM: A scoring system for perforative peritonitis. J Clin Diagn Res.

[32] Ngulube A, Muguti GI, Muguti EG (2019). Validation of POSSUM, P-POSSUM and the surgical risk scale in major general surgical operations in Harare: A prospective observational study. Ann Med Surg (Lond).

